# Saliva Sampling for Prospective SARS-CoV-2 Screening of Healthcare Professionals

**DOI:** 10.3389/fmed.2022.823577

**Published:** 2022-01-26

**Authors:** Adalbert Raimann, Alex Farr, Mercedes Huscsava, Wilfried Krois, Robert Strassl, Julia Schellnegger, Fabian Eibensteiner, Bernadette Göschl, Hannah Schned, Philipp Steinbauer, Mathias Hetzmannseder, Fabian Stiegner, Susanne Greber-Platzer, Herbert Kiss, Paul L. Plener, Christoph Aufricht, Angelika Berger, Michael Wagner

**Affiliations:** ^1^Division of Pediatric Pulmonology, Allergology and Endocrinology, Department of Pediatrics and Adolescent Medicine, Comprehensive Center for Pediatrics, Medical University of Vienna, Vienna, Austria; ^2^Division of Obstetrics and Feto-Maternal Medicine, Department of Obstetrics and Gynecology, Comprehensive Center for Pediatrics, Medical University of Vienna, Vienna, Austria; ^3^Department of Child and Adolescent Psychiatry, Comprehensive Center for Pediatrics, Medical University of Vienna, Vienna, Austria; ^4^Department of Pediatric Surgery, Comprehensive Center for Pediatrics, Medical University of Vienna, Vienna, Austria; ^5^Division of Clinical Virology, Institute of Laboratory Medicine, Medical University of Vienna, Vienna, Austria; ^6^Division of Pediatric Nephrology and Gastroenterology, Department of Pediatrics and Adolescent Medicine, Comprehensive Center for Pediatrics, Medical University of Vienna, Vienna, Austria; ^7^Division of Neonatology, Pediatric Intensive Care and Neuropediatrics, Department of Pediatrics and Adolescent Medicine, Comprehensive Center for Pediatrics, Medical University of Vienna, Vienna, Austria; ^8^Comprehensive Center for Pediatrics, Medical University of Vienna, Vienna, Austria; ^9^Department of Child and Adolescent Psychiatry and Psychotherapy, University of Ulm, Ulm, Germany

**Keywords:** COVID-19, healthcare provider, saliva, SARS-CoV-2, screening

## Abstract

**Objective:**

The objective of this study was to analyze the feasibility and acceptance of a non-invasive, daily and proactive screening program for SARS-CoV-2 infection employing serial saliva testing, in combination with a digital questionnaire among healthcare providers (HCPs) in a multi-professional setting.

**Design:**

This was a prospective cohort study involving HCPs from different units at a single tertiary care center, over a pilot phase of 4 weeks during the first wave of the COVID-19 pandemic from April 18th to June 6th, 2020.

**Setting:**

Pediatric tertiary patient care units, Comprehensive Center for Pediatrics, Medical University of Vienna.

**Subjects:**

HCPs from different units, including physicians, nurses, midwives, and administrative staff (with patient contact) were considered eligible for the study. Study participants were working in different settings in our center at varying levels of risk exposure.

**Interventions:**

Saliva collection from mouth gargle and electronic symptom and exposure monitoring (eSEM) was performed by participants at the onset of each regular clinical shift (day or night shift), using an anonymous ID for matching the results.

**Measurements:**

RT-PCR of all saliva samples, eSEM, as well as feasibility and acceptance thereof.

**Results:**

Two hundred and seventy-five volunteers collected 1,865 saliva samples and responded 1,378 times in the eSEM during a 4-week period. 1,331 (96.7%) responses were that the testing was feasible and acceptable. The most common severe symptom during the 4-week period mentioned by HCPs was headache, reported 54 times (3.9%). Two SARS-CoV-2 positive samples—one of them being associated with symptoms—were identified. The acceptance rate among HCPs was 96.6%.

**Conclusion:**

Serial saliva screening was a well-accepted and feasible method for monitoring SARS-CoV-2 infectious state in health care professionals. Combination of regular SARS-CoV-2 tests with sequential saliva collection and storage could potentially represent a highly efficient strategy to identify and trace virus positive staff for employee and patient safety.

## Introduction

Severe acute respiratory syndrome coronavirus type 2 (SARS-CoV-2) infections as well as many other concomitant health care issues caused by the coronavirus disease 2019 (COVID-19) pandemic pose a burden on healthcare systems worldwide (1). High rates of asymptomatic carriers (2, 3) and limited large scale screening capacities display imminent risks for healthcare providers due to (i) healthcare professionals (HCPs) being quarantined for symptomatic infections or contact to SARS-CoV-2 positive individuals, and (ii) exposure of vulnerable patient cohorts at risk for severe forms of COVID-19 to asymptomatic carriers (HCPs and patients).

The limited availability of testing material in combination with a high patient volume lead to restricted screening capacities for HCPs during the ongoing COVID-19 pandemic (4, 5). The effectiveness of novel screening approaches to reduce subclinical SARS-CoV-2 infection at healthcare facilities is a matter of ongoing research (6, 7). Implementation of a practical screening method with self-performed material collection, in combination with digital assessment tools, could increase patient and staff safety via early detection of possibly infected but asymptomatic carriers.

The current gold-standard to detect SARS-CoV-2 in humans is real-time reverse transcription polymerase chain reaction (RT-PCR) (8, 9). Samples are most commonly collected via nasopharyngeal or oropharyngeal swabs. These methods are well-established among primary and tertiary care centers, as well as COVID-19 testing centers. Nevertheless, the invasive nature of sampling imposes discomfort and limited acceptance, especially in long-term screening programs (10).

Recent studies have explored the use of saliva as a potential material for detecting SARS-CoV-2 instead of using naso- and oropharyngeal swabs, appearing to overcome limiting factors of the current swab techniques. The use of saliva for SARS-CoV-2 screening shows benefits regarding the acceptance and comfort of the screened individuals, as well as optimization of healthcare resources (11–15). Saliva has proven to be comparable to the common swab techniques, with numerous studies (16–19) describing high sensitivity and specificity for the detection of SARS-CoV-2 by RT-PCR.

In the present study, we evaluated the feasibility and acceptance of a non-invasive screening program for SARS-CoV-2 infection, using daily saliva collection with consecutive RT-PCR analysis, in combination with a digital questionnaire among HCPs in an interdisciplinary tertiary care center. We hypothesized that the combination of regular SARS-CoV-2 PCR tests with serial saliva collection could represent a highly efficient strategy to identify and trace virus positive staff for employee and patient safety and that this program might be a well-accepted and low-resource screening strategy for early detection of SARS-CoV-2 outbreaks in a healthcare setting.

## Materials and Methods

### Study Design

The current study was a prospective cohort study for the collection of serial saliva samples in order to detect SARS-CoV-2 infection in HCPs from differently exposed units in a single tertiary care center, in combination with a digital questionnaire for thorough symptom screening, over a pilot phase of 4 weeks during the first wave of the COVID-19 pandemic in Austria (i.e., April 18th to June 6th, 2020).

### Aims of the Study

The primary endpoints of the study were feasibility and acceptance of daily proactive non-invasive screening for SARS-CoV-2 infections, as measured by electronic symptom and exposure monitoring (eSEM), as well as the number of SARS-CoV-2 positive saliva samples. Secondary endpoints were to establish a digital tool to assess individual symptoms on a daily basis, to characterize the most common symptoms exhibited by HCPs working in a pediatric tertiary care center during the first wave of the COVID-19 pandemic.

### Participants

All HCPs (i.e., physicians, nurses, midwives, administrative staff with patient contact) were invited to participate in the study. Inclusion criteria were employment at the Comprehensive Center for Pediatrics (CCP) of the Medical University of Vienna, which integrates the largest perinatal center in Austria. Study participants were working in different hospital settings with different risk exposition, as follows: (i) low risk exposition (e.g., intensive care), (ii) intermediate risk exposition (e.g., contact with non-febrile or asymptomatic patients), (iii) high risk exposition (e.g., contact with febrile or symptomatic patients). All participants gave written informed consent for the use of their anonymized data and sample collection for further analysis. The study was approved by the local institutional review board (IRB number: 1344/2020) and the local data protection committee.

### Setting

Data of all study participants were anonymized to non-retractable study IDs. Animal and plant names were used as ID mnemonics for participants to increase identification and adherence to the study. Baseline characteristics (sex, age in decades, weight) were collected at study entry. Saliva collection and eSEM were performed by participants at the start of each regular clinical shift (day or night shift) using the initial anonymous ID for matching of the results. As the stability of SARS-CoV-2 RNA in saliva is already proven (20) and the emphasis of this study was to prove feasibility and acceptance of the new sampling method, the decision was made to include not only early morning saliva samples but also nocturnal samples. Instructions for saliva collection were easily accessible at a central study point in each participating unit. Participants were informed not to eat, drink, or consume water right before sampling.

### Material and Storage

Saliva samples from mouth gargle were collected in 2 ml tubes through a regular 200 μl pipette tip being able to pass saliva but incompatible with mucous sputum probes. Samples were collected at the same day and stored at −80°C for further processing and common analysis after the study period, i.e., the extraction of the SARS-CoV-2 RNA as described in 2.6 RNA isolation and quantification.

### RNA Isolation and Quantification

**Figure 1:**
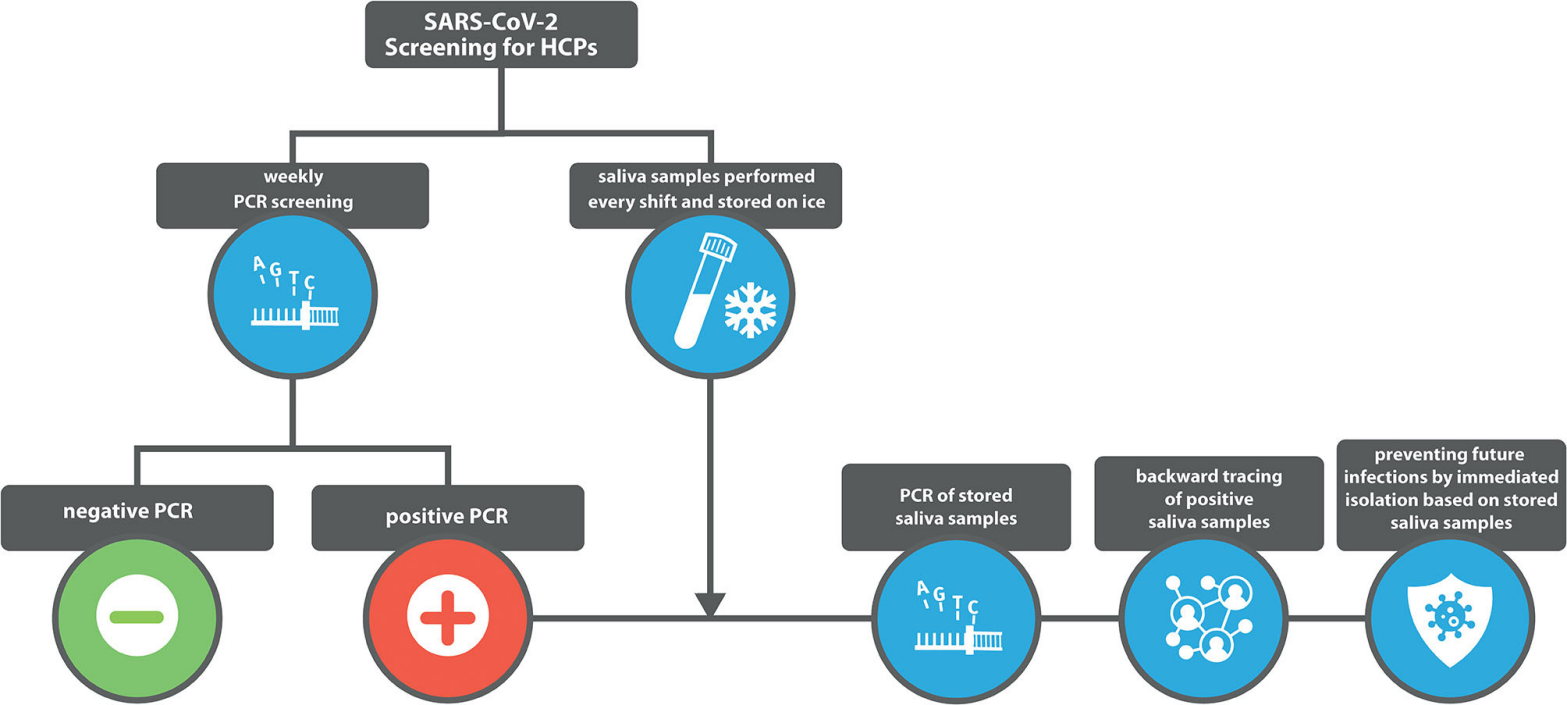
Schematic overview of the basic study concept.

Coronavirus SARS-CoV-2 RNA was extracted from nasopharyngeal as well as saliva samples using the Perkin Elmer Chemagic 360 system (chemagic™ Viral DNA/RNA 300 Kit, Perkin Elmer, Waltham, Massachusetts, United States). RT-PCR was carried out on a Roche Lightcycler 480II platform using primer/probes according to the protocol published by Corman et al. (21). To be conform to the ethical and data protection presets defined at study inception, analyses were performed after the phase of sample collection. In this preliminary study, nasopharyngeal RT-PCR was mandatory for all HCPs on a weekly basis and as a prove of concept, all collected saliva samples were additionally analyzed by RT-PCR. As shown in Figure 1, the concept of this study in a real-world setting is that the additionally stored saliva samples will only be tested for backward tracing and prevention of future infections if the weekly nasopharyngeal RT-PCR is positive.

### Electronic Symptom and Exposure Monitoring (eSEM)

Acceptance of screening, individual symptoms (e.g., fever, coughing), and exposure (e.g., the number of extramural activities) were retrieved anonymously on a daily basis at the beginning of regular clinical shifts. Participants were asked to complete a digital questionnaire using SurveyMonkey (www.surveymonkey.com, SurveyMonkey, San Mateo, California, USA), which was accessible on mobile phone or computer. Collected data was immediately linked to the anonymous study-ID and date of data entry. Information on the routine SARS-CoV-2 PCR testing state, which was performed on a weekly basis by every HCP according to local hospital guidelines, was retrieved from all participants for the preceding 2 weeks, again linked with the anonymous study-ID.

The questionnaire consisted of 15 questions. A total of eight questions was designed to assess information about acceptance of the studied screening method on a 3-Point-Likert-Scale by assessing the degree of discomfort imposed by the daily sampling technique (“no problem for me,” “a little uncomfortable,” “uncomfortable”), the occurrence of individual symptoms on a 3-Point-Likert-Scale (“yes,” “a little,” “no”), one question assessed the occurrence of fever on a 3-Point-Likert-Scale (“yes, measured,” “yes, feels like it,” “no”), four questions retrieved dichotomous (“yes,” “no”) information on SARS-CoV-2 exposure, and one question was designed to assess frequency of risk mobility for necessary supplies (groceries) on three different levels (“ <2 times per week,” “3–5 times per week,” “>5 times per week”).

### Statistical Analyses

Categorical variables were calculated using absolute and relative frequencies. Continuous variables were either calculated as mean and standard deviation (SD), or as median and interquartile range (IQR), depending on data distribution. Per convention, the level of significance was set at *p* < 0.05 (two-sided). Data were analyzed using SPSS version 26.0 (IBM Corp., Armonk, NY).

## Results

**Figure 2:**
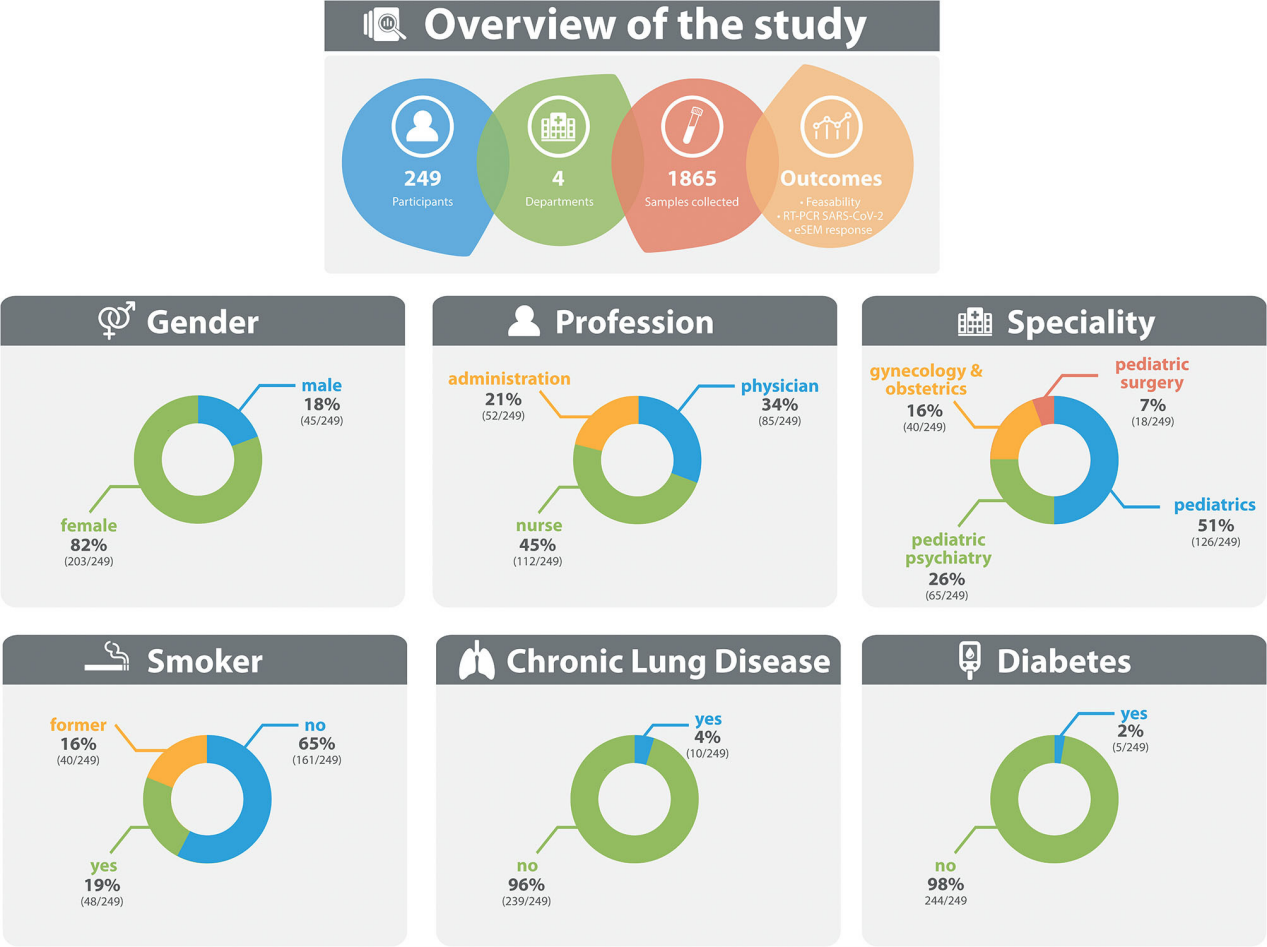
Overview of participant characteristics.

During a period of 4 weeks, we were able to collect a total of 1,865 saliva samples from 275 participants. On average, 6.78 saliva samples were collected from each HCP. Two hundred and forty-nine participants (90.5%) provided voluntary epidemiologic information including sex, profession, and risk profile. 203 (81.5%) participants were female. 45.0% (112/249) were categorized as nursing staff, 34.1% (85/249) as medical doctors and 20.9% (52/249) as administrative staff. 64.3% (160/249) were non-smokers, 19.3% (48/249) active and 16.1% (40/249) former smokers. 4.0% (10/249) stated an existing lung disease, 2.0% (5/249) diabetes mellitus (Figure 2).

### Primary Outcome

To evaluate feasibility and acceptance, we asked participants if they were comfortable with this kind of daily routine testing. 1,331 (96.6%) responses out of 1,378 in total were that the testing was “*no problem*,” 42 (3.0%) were that it was only “*slightly uncomfortable*” and only five (0.4%) expressed that the procedure felt “*uncomfortable*” (Table 1). All saliva samples were analyzed by RT-PCR in bulk after collection of all samples. Two detected (0.1%) samples were positive for SARS-CoV-2 in the RT-PCR, however the CT value was >35 in both cases. As every sample was marked with the study ID and date, it was possible to trace the two samples. One participant did not show any symptoms in eSEM, while the other participant reported muscle ache in the eSEM 10 days after the positive sample. Due to the low number of positive samples, intended statistical analyses and comparisons with self-declared symptom questionnaires were not performed. As every sample was marked with the study ID and date, it was possible to trace the two samples.

**Table 1:** Electronic symptom score data.

	***N* (total = 1,378)**	**Percentage**
Do you have a fever today (>37.5°C)?		
Yes, measured	2	0.1
Yes, feels like	1	0.1
No	1,375	99.8
Do you have to cough today?		
Yes	8	0.6
A little	44	3.2
No	1,326	96.2
Do you feel tired today?		
Yes	34	2.5
A little	143	10.4
No	1,202	87.2
Do you have heavy breathing today?		
Yes	3	0.2
A little	16	1.2
No	1,359	98.6
Do you have muscle pain/body aches today?		
Yes	13	0.9
A little	65	4.7
No	1,300	94.3
Do you have a headache today?		
Yes	54	3.9
A little	123	8.9
No	1,201	87.2
Do you have a sore throat today?		
Yes	12	0.9
A little	67	4.9
No	1,299	94.3
Do you have a cold today?		
Yes	32	2.3
A little	110	8.0
No	1,236	89.7
Do you have the feeling that your taste/smell is different?		
Yes	2	0.1
A little	10	0.7
No	1,366	99.1
Is someone in your household quarantined due to contact with a SARS-CoV-2		
positive person?		
Yes	18	1.3
No	1,360	98.7
Did someone in your household show any of the symptoms mentioned above?		
Yes	47	3.4
No	1,331	96.6
Did you spend time abroad the last 2 weeks?		
Yes	13	0.9
No	1,365	99.1
Is/was anyone in your household ever tested positive for SARS-CoV-2?		
Yes	3	0.2
No	1,375	99.8
How often did you leave your home for necessary supplies during the last week?		
<2 × /week	248	18.0
3–5 × /week	507	36.8
>5 × /week	623	45.2
What is your experience with the daily sampling?		
No problem	1,331	96.6
Slightly uncomfortable	42	3.0
Uncomfortable	5	0.4

Saliva sampling took ~90 s, the questionnaire around 30 seconds.

### Secondary Outcomes

We received a total of 1,378 eSEM responses. The detailed results are displayed in Table 1.

#### Self-Declared Symptoms

The most common severe symptom mentioned by HCPs was headache, reported by 54 responses (3.9%).

Only two responses (0.1%) reported fever above 37.5°C and one reported to feel fever without measurement during the 4-week period. Three (0.2%) responses reported severe dyspnea, while 16 (1.2%) reported mild dyspnea. Eight responses (0.6%) reported severe cough and 44 (3.2%) mild coughing symptoms. Rhinitis was also a common symptom with 32 responses (2.3%) indicating heavy rhinitis-associated symptoms and 110 responses (8.0%) declaring mild cold symptoms.

#### Exposure

Forty-seven (3.4%) responses reported symptoms such as headache, coughing, and fever in housemates, during the study period.

Three responses (0.2%) reported to live with a quarantined housemate for 2 weeks due to exposure to a COVID-19 positive individual.

Most answers reporting symptoms in the questionnaire [623/1,378 (45.2%)] stated that necessary items were purchased more than 5 times per week, whereas 248 (18.0%) reported purchasing necessary items <2 times per week and 507 (36.8%) reported a 3–5 times grocery shopping routine per week.

### Screening Costs

Screening material was based on availability despite logistic burdens during the pandemic, and consisted of 2 ml tubes, 2 μl pipet tips and 10 ml vials of NaCl 0.9%. The raw material costs for saliva screening material remained below the targeted 0.5 €/ sample.

## Discussion

This prospective cohort study analyzed feasibility of a new methodology for longitudinal SARS-CoV-2 screening of asymptomatic staff at pediatric and obstetric tertiary care units with self-collected saliva samples combined with a digital symptom and scoring tool, during the first wave of the COVID-19 pandemic.

The establishment of an economically justifiable and well-accepted strategy for screening of HCPs, as presented by the tools evaluated in this study, represent methods capable of optimizing ongoing screening regimes for an easy improvement of temporal infection detection and staff safety.

While many healthcare providers established screening strategies for HCPs, economic and organizational burdens commonly impede “screen as you work” monitoring schemes. Thus, HCPs are commonly screened on a weekly basis. Since the development of rapid antigen tests, allowing test results within 15–20 min, many screening regimens use PCR testing as second line testing despite markedly lower sensitivity of rapid antigen tests (22, 23). While sensitivity of SARS-CoV-2 detection in asymptomatic individuals is a challenge for all sampling techniques, saliva sampling has also been reported to be more sensitive than nasopharyngeal or nasal swabs (24). Saliva sampling should follow standardized protocol, but at the time of initiation of the study, no such protocol was available since this method was fairly new.

Our proposed non-invasive serial screening approach was easily implemented and highly accepted among HCPs participating in this study, where 96.6% felt comfortable with regular saliva screening and daily digital symptom scoring.

**Figure 3:**
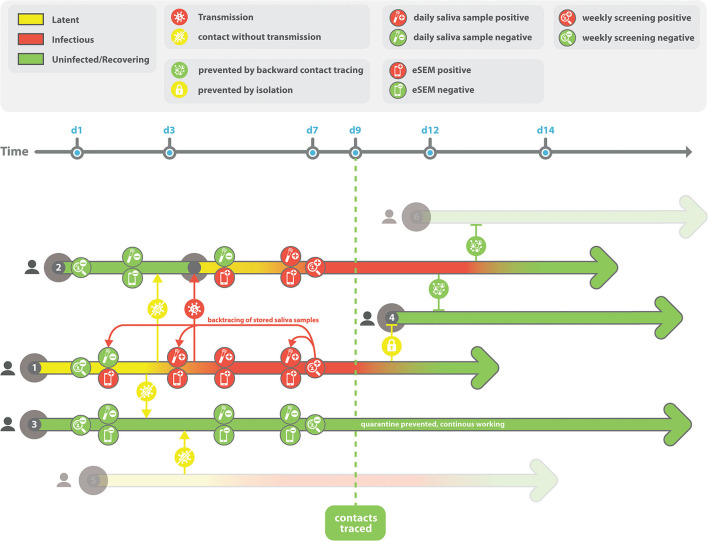
Schematic depiction of a saliva-based screening strategy for healthcare professionals.

Based on the high technical and financial feasibility, we propose a testing strategy based on serial saliva sampling as performed in this study: In the case of positive testing or proven infection status of an HCP, stored samples of the previous working days could be tested retrospectively to identify the first day of SARS-CoV-2 positivity at work. As shown in Figure 3, HCP1 develops minor symptoms on day (d) 1 but is negative in the regular weekly screening. On d3, infection of another HCP at work occurs. On d7, HCP1 is tested positive and quarantined. Daily collected saliva samples are analyzed to identify the first day with a positive sample to help backtracing of other contacts. HCP2 becomes infected by HCP1 on d3, reaching contagiousness around d5. Saliva samples prove negativity at d4 and prevents isolation of contacts on this day. Positivity could be shown on d5, preventing future infections by immediate isolation based on analysis of stored saliva samples. HCP3 has non-contagious contact to HCP1 on d1, with no further close contacts. Due to negativity of HCP1 at d1, lack of contact and analysis of stored saliva samples, quarantine is prevented and HCP3 can continue working. HCP4 would have had future contacts to HCP1 and HCP2. Potential infection was prevented by quarantine of HCP1 based on weekly routine screenings and by isolation of HCP2 based on infection backtracing in stored saliva samples. A retrospective screening of all contact HCPs of the same unit would be possible in a fast and efficient manner without the need for *post-hoc* recruitment of potentially contagious individuals for retesting. Further, questionnaire data of the index HCP and contact HCPs could facilitate the determination of symptom onset and retrospective evaluation of clusters or outbreaks in single units or across different departments. Thus, the proposed screening strategy could serve in addition to established screening programs to allow tracing of SARS-CoV-2 positivity in health care settings to limit the risk of spreading as well as HCP quarantine.

In this pilot study, saliva collection, electronic monitoring and retrospective analyses were performed on a voluntary basis to evaluate feasibility and acceptance among HCPs. Participation rates have been estimated around 50–75 %, motivation was very high among the participating division. However, an exact number of participation and refusal rate could not be included due to first, the mandatory anonymous study design which does not allow to work on stuff lists, and second, numerous staff rotations due to preparation for the pandemics hindered to calculate a reliable, absolute count of HCPs being in charge at participating divisions. While analyzing 1,865 individual saliva samples from 275 participants, only two participants had samples positive for SARS-CoV-2 by PCR, whereas we have received a total of 216 responses indicating to suffer from fever, severe or mild dyspnea, severe cough or mild coughing symptoms, heavy rhinitis, or mild cold symptoms. HCPs are not only exposed to SARS-CoV-2 but, especially, in pediatrics, to multiple other infectious conditions. The number of positive PCR tests was low, as the total number of Austrian numbers stayed on a very low level as this was the first country in Europe undergoing a total lockdown. The low numbers are in line with the lack of reports on positive result in the—obligatory—weekly nasal swab testing for all HCPs. Further, no cluster developed during that wave in our clinic. Thus, we are very sure, that the HCPs reporting mild symptoms did not suffer from COVID 19 and results might have been differently a few months later or especially nowadays. These surprisingly low positive results in our study are moreover in line with a recently published study from the Netherlands, where a total of 1,796 HCPs from three different hospitals were screened with RT-PCR from naso- and oropharyngeal swabs during the first wave of the COVID-19 pandemic, resulting in a SARS-CoV-2 positivity rate of 5% (25). Furthermore, several recently published studies on seroprevalence of SARS-CoV-2 antibodies among HCPs confirm these finding (26, 27). In one study, conducted during May and July 2020, only 0–17% of pediatric HCPs displayed IgG against SARS-CoV-2, with a seroprevalence of <2% in continental Europe, in comparison to 17% in the United Kingdom. The respective multi-center study was also performed in our hospital, with 0% of participants having IgG antibodies, which could explain the low rate of positive PCR results in our study. As recently discussed by Goldblatt et al., such results may originate from successful health care facility mitigation measurements, or may stem from lower nosocomial exposure and/or lower transmission rates of SARS-CoV-2 from infected children to adults (26). A total of five out of 1,378 (0.4%) responses by participants expressed uncomfortableness with this procedure. The discomfort reported 5 times during the study period could not be further analyzed due to data protection regulations, therefore, not knowing whether the mentioned responses were entered from the same individual or if the same HCP reported “*no problem*” when being previously tested. Communication with the participants in general revealed that the salty taste of the saline solution was negatively perceived a few times. Since participation in this study was voluntary, our results might be biased by the use and acceptance of the program by generally more digital-native participants. Therefore, general acceptance rates might be lower, especially regarding the digital symptom scoring system. Though, digital symptom scoring has proven to be of great value, especially when gain of knowledge about SARS-CoV-2 is crucial to keep up with current dynamics in the worldwide pandemic and to adapt screening management in real time (28).

The most severe symptom reported was suffering from headache, but due to privacy guidelines, we have not been able to identify the individuals reporting this symptom, therefore we were not able to ask the affected HCPs for a specific reason, e.g., chronic migraine. According to recent studies about acceptability of saliva sampling, as already mentioned in the introduction, and in our study 96.7 % indicating that this testing was feasibly and acceptable, the reason for the reported headache is not likely to be associated with the testing method.

Most interestingly, almost half (45.2%) of HCPs displayed risky exposure behavior by leaving their home more than five times per week for necessary supplies, while 18.0% stated to do this <2 times per week only. This is important since it should lead health care organizations to promote prudence among their HCPs regarding mitigation measures especially outside the healthcare setting. Evidence for these suggestions comes from an international study where multiple exposures of HCPs outside the healthcare setting were strongly associated with SARS-CoV-2 infection (29).

Regarding the economic advantage of this testing technique, storage is probably the most critical position in this approach, as high numbers of samples are collected. Thus, this approach relies on the availability of a storage facility. With limiting the number of days of conservation to 14 and use of small sampling tubes such as 1 ml tubes, the costs are around 20 cents to 1 euro per sample. Compared to the costs of increased number of HCPs in quarantine or nonselective RT-PCR screening approaches, we see a huge advantage in the given approach. Furthermore, we would propose to use this broadly accepted saliva sampling in a “store and trace” approach to reduce costs while still having an effective approach. This is especially important as internationally regular PCR are increasing as well as costs, respectively.

Since it remains unclear if transmission of SARS-CoV-2 can be reduced or prohibited by the recently approved SARS-CoV-2 vaccine (30), routine testing of HCPs and patients for SARS-CoV-2 will remain an important aspect of infection control, especially in the care of high-risk patient groups. Additionally, with upcoming new variants of the Coronavirus SARS-CoV-2 spreading even faster than the original virus the number of cases in Austria and also worldwide are currently still rising and the contact tracing is completely overworked. Furthermore, non-selective RT-PCR tests from all healthcare providers are performed on a regular basis, which is very cost-intensive. If RT-PCR testing would be decreased from 2-3 times a week to one time a week and daily saliva samples would be stored and only analyzed if RT-PCR from a nasal swab is positive, this would allow for a concrete back tracing and quarantine as well as additional RT-PCR sampling of saliva samples of all contact persons. In light of this, our proposed approach of routine SARS-CoV-2 screening of saliva conjointly with digital symptom tracking remains a potentially useful tool for identification and tracing of virus positive staff throughout the further course of this pandemic. Prospective screening was highly accepted by HCPs, with a complete acceptance rate of 96.6%, and could be safely implemented with easily available consumables, and can be done by HCPs themselves.

## Conclusion

Serial saliva screening is a feasible, well-accepted and convenient method for monitoring of SARS-CoV-2 infections in HCPs. Combination of regular SARS-CoV-2 tests with sequential saliva collection and storage could represent a highly efficient strategy to identify and trace virus positive staff with subsequent reduction of staff and patient exposure as well as need for quarantines and sick leave, potentially limiting the impact of COVID-19 on stressed health care systems.

## Data Availability Statement

The raw data supporting the conclusions of this article will be made available by the authors, without undue reservation.

## Ethics Statement

The studies involving human participants were reviewed and approved by the Local Institutional Review Board (IRB number: 1344/2020) and local data protection committee of the Medical University of Vienna. The patients/participants provided their written informed consent to participate in this study.

## Author Contributions

AR and MW conceptualized and designed the study and drafted the initial manuscript. AR, MW, AF, MH, WK, RS, JS, FE, BG, HS, PS, MH, and FS designed the data collection instruments, collected data, carried out the initial analyses, and reviewed and revised the manuscript. SG-P, HK, PP, CA, and AB helped interpreting the results and reviewed and revised the manuscript. All authors approved the final manuscript as submitted and agree to be accountable for all aspects of the work.

## Funding

This study was funded with support from the Comprehensive Center for Pediatrics (CCP) Starter Grant and the Scientific Fund of the Mayor of Vienna (Project ID: COVID029).

## Conflict of Interest

The authors declare that the research was conducted in the absence of any commercial or financial relationships that could be construed as a potential conflict of interest.

## Publisher's Note

All claims expressed in this article are solely those of the authors and do not necessarily represent those of their affiliated organizations, or those of the publisher, the editors and the reviewers. Any product that may be evaluated in this article, or claim that may be made by its manufacturer, is not guaranteed or endorsed by the publisher.
